# Porcelain Dental Art

**Published:** 1895-04

**Authors:** W. A. Capon

**Affiliations:** Philadelphia.


ARTICLE VI.

### PORCELAIN DENTAL ART.

W. A. CAPON, D. D. S., PHILADELPHIA.

There is probably no subject now exciting greater interest in our profession than porcelain as a substitute for unsightly and unreliable operations, whether it be a portion or whole tooth, or for several teeth in form of a bridge, or of a partial denture. This class of work presents many ways of overcoming disagreeable deformities and defects in an artistic and acceptable manner, both to patient and operator, enabling us to reach a perfection long desired in dental art. The experimental stage is passed, for the work has proven reliable and satisfactory under the most discouraging conditions. A few facts may be acceptable.

The requirements for success are many, and those who have given this subject some consideration will be better able to appreciate this fact than those who have yet to make their first attempt.

Porcelain dental art embraces three distinct classes of work: Fillings, crowns and bridges. Fillings are the most difficult, more difficult than any other kind of filling in use, because mechanical art and science are combined in one little operation. A porcelain filling properly made and adjusted will be almost unnoticeable even to the professional eye. Being held in position more by adhesion of cement than by mechanical means, it adds strength to a frail tooth, and is also a non-conducting material, which is an important consideration when operating near the pulp. This filling is especially indicated in labial and large proximal cavities and contoured tips, and very desirable in broken centrals, or where excessive abrasion has caused much loss of tooth. In such, the use of gold means a long, tedious operation, and always conspicuous, attracting observation to the defect instead of hiding it, and very frequently the tooth structure is such that plastic work is almost imperative.

Preparation for this work consists of enough separation to allow of drawing the impression from the cavity without change of form and the insertion of filling with freedom, and proper excavation of cavity which others, inasmuch that no undercut is made till after the filling is made; also making the edges square, avoiding the use of sandpaper, or the filling will have an overhanging edge, making a coarse joint by retaining cement. The platinum foil used for impression is gage .001, or about 50, and is first thoroughly annealed by placing it in a furnace and giving it heat required. For fusing porcelain, the object of using the furnace is to protect from the gas flame, which insures the softness necessary for a good impression. If this point is not observed there will be a spring in the metal that will prevent burnishing to walls and edges of cavity. Rubber tips and amalgam burnishers of various sizes are used to take impression, using care not to have a fold on the edge, which is especially liable in large proximal work. Unless the cavity is very shallow the impression will have very little bottom, but this is of no importance and cannot be avoided. This is easily remedied by using care not to force the porcelain in the matrix too far. Gently tapping the edge of the mold will settle the porcelain and give it a smooth, wet surface. The excessive moisture is withdrawn by laying the work on a clean napkin or bibulous paper; then carefully dried by heat in the furnace, which is gradually increased, till sufficient for fusing. This part is called first baking or biscuiting and, while it is not particularly necessary to have a glossy surface, that fact will show that maximum shrinkage has taken place. The filling is now placed in cavity and edges thoroughly burnished to the tooth, which are always drawn from original form by contraction of porcelain. This will be more noticeable in large fillings because of smaller surface of platinum to resist the spheroidal tendency of the porcelain while in a molten state.

The filling is now ready for finishing, and it is just at this stage of the work where it differs so materially from any other filling, because it is being made out of the mouth and away from the patient; only practice and memory can be a guide as to the proper shape, shade and quantity of porcelain required for correct work, allowance for a skrinkage of fully a fifth must be made and care taken not to have an overhanging edge, or the joint will be coarse. A filling can be baked several times, if the porcelain is good; otherwise repeated heating will effect both shade and contour. After the platinum is removed, it cannot be put in the heat again. When the filling is finished, the platinum is gently pulled off by means of small pointed pliers, commencing at edge of filling and working to the middle, which will leave edges unbroken.

Undercuts are now made with diamond or thin hard rubber and corundum disks, and filling cemented in position. It is very necessary that large tips and corners should have platinum pins or wire loops, these make the work difficult and tedious, as soldering is avoided, if possible, on account of its effect on such a small body of porcelain. A well-made filling will have the graded shades of the natural tooth; also the proper density and gloss with edges nicely fitting cavity line, allowing for little grinding, as polishing discolors the cement line and takes a long while to become clean. The cement must be one of medium speed in setting, and mixed to a creamy consistency, so that very little pressure is required to put the filling into position. If cement is unyielding; or sets too rapidly, immediate withdrawal is recommended, or the results will be unsatisfactory. Many dentists make this mistake, not only with this work but in placing crowns and bridges, forgetting that cement mixed beyond a proper thickness loses its tenacity, which is the quality required.

After removing excess cement, hot air should be applied, and the operation finished by covering the whole work with paraffin wax, chloro percha, sandarac or rubber varnish, leaving trimming till a later sitting, when the cement will be thoroughly hard.

One of the principle assertions used as an argument against this work has been the solubility of the cement to such an extent as to loosen the filling. Theoretically, this would seem probable; but, after years of practical tests in all sorts of mouths, and under all conditions, the cement remains firm if the joints are good. The use of rubber-dam is not particularly advantageous when doing this work, unless in some cases where long dryness is particularly necessary; and under no consideration should it be used till the filling is complete and ready for insertion, as the dry tooth will be far from its natural shade.

Molar fillings are to be avoided, unless very accessible, because of the difficulty in getting a proper impression; and especially so on masticating surfaces, where the filling must be particularly strong and tight to withstand the force required in chewing.

Pinhead and shallow labial cavities are very tedious; and frequently the shade is entirely destroyed by the cement. However, time will remedy this defect very considerably, for porcelain fillings improve in appearance by age.

The shade of tooth is obtained by numbered buttons, corresponding with bottles of porcelain body of same number, and may range in quantity of shades from one dozen to two dozen. My assortment consists of twenty, and of different manufactures, but all of the same standard, and requiring the same heat necessary for continuous gum work, which is considerably less than "block body," or that used for manufacturing teeth.

Cleanliness must be observed in using porcelain and cinders, plaster or dust of any kind carefully kept from the mixing slab; while the water should be perfectly clean—and, if possible, use distilled or filtered rain water.

One of the most important points to be considered in the baking of porcelain is the furnace. Every dentist is familiar with the coke or coal oven for continuous gum work, but comparatively few are informed on the newer furnaces adapted for smaller work, such as crown- or bridge-work. They consist of open flame and fire clay, or platinum muffles. Fire clay is preferable on account of purity of color and less tendency to gas the porcelain, but requires more time for heating than those made of platinum, while the latter are more convenient for quick and small work, and a heat of 3,300° F. can be obtained in from three to five minutes from time of lighting. The most scientific and perfect of all furnaces is the recent invention of Dr. H. C. Land, of Detroit, Mich., it requires no forced draft, and can be used with refined oil, crude petroleum, gasoline, or natural or illuminating gas. All furnaces, whether for coke, gas or oil, should be capable of a heat not less than 3,000° F., as true porcelain requires that to insure color and strength.

The greatest care and vigilance is required by the most experienced porcelain workers to prevent "gasing," for, as has been truly said, "during the process of combustion in furnaces, whether the fuel is anthracite, coke or gas, carbon in the form of decomposed hydrocarbon, or smoke or carbonic oxid, may be present in varying proportions, according to the amount of air passing through the combustion chamber, and in one or both forms is extremely liable to penetrate to the interior of the muffle, here to combine with the oxygen contained in the porcelain to form $CO_2$ bubbles, thus the body and coloring matter become deoxidized, making the mass porous." When this occurs the color will vary from white to a dull grey, according to the amount of carbon entering the muffle. Sometimes the discoloration is very slight, and would pass the unexperienced eye as being correct till compared with a perfect baking. It is useless to attempt a remedy by patching or covering parts affected; those portions should be destroyed and pure material added and fired again till satisfactory, for a better jet can be made and shade and shape quite equal to what can be bought, and the whole operation consuming very little more time, and a last but a very important part is the fact that you have created a more favorable impression with the patient, for in no branch of dentistry is there a better opportunity to do a little legitimate advertising.

This system covers various kinds of crowns, offering many means of overcoming difficult cases, enabling the ingenious dentist to make or carve one or more teeth to any shape to fit any peculiarity. There are pin crowns, with or without collars, to fit necks of roots. Crowns with tubes to fit over posts, screwed into the canals and crowns of solid porcelain, slightly concaved, to be used with pin or post. But the most useful of all kinds is the jacket crown, which I will describe and illustrate later on. The universal use of "store" crowns, either pin or tube, can be attributed to the fact that little skill or time is required to adjust them, and, when nicely mounted, look very natural; but there are many places where a ready-made crown cannot be used to the best advantage, and then a knowledge of porcelain, with the proper appliance, will prove very valuable, saving much time and annoyance to the patient, and after some practice it will be unnecessary to buy a crown for any work.

In making a banded porcelain crown, the first preparation is similar to that of a Richmond, excepting that the metal used is entirely platinum and the plate tooth should have no backing, the pins extend to the post, which is left projecting through the cap to allow of soldering, thereby holding facing in position and porcelain added till the desired shape is obtained. A crown without band is made in the same manner, though in either case a pin tooth is not strictly necessary, for thin veneers are used with the advantage of less danger to check while heating. When veneers are used a foundation of porcelain must be made first, so that veneer will be held in position more easily. Having a proper quantity of biscuited body on the crown will also prevent the veneer from falling out of position by shrinkage.

A tube crown is made somewhat different, as the first proceeding is to thread the canal, and use a post of the home pattern, or something similar. The end of the screw should be dipped in thin cement and then turned into place, allowing excess cement to cover face of root, and amalgam packed over that surface and surround the screw, thus making a very strong and almost indestructible foundation. After this has become sufficiently hard, prepare for the crown by making a platinum tube to fit the projecting post in the following manner: Take a spare post, the same as that already used, and twist platinum foil about it, which will form the tube exactly, then take a small piece of the same foil of sufficient size to cover the root, and puncture a hole in the center and slide over the tube already on the post; draw it off carefully, and solder with the smallest possible quantity of pure gold. This being done, there will be a platinum affair that will cover tube and face of rcot, which only requires burnishing around edges and is ready for the porcelain body. Place thick porcelain paste around tube and lay veneer in its place; then carefully draw the whole from position, and lay it on some absorbing material till the excess of moisture is withdrawn. Put this in the [illegible]rnace and gradually heat till biscuited. To complete the crown, place it in position and thoroughly burnish the gum line; then trim of excess of platinum and add body wherever required. After finishing, it is optional whether the platinum is left remaining or not, but the front portion is generally removed for fear of showing metal line. Veneers are generally used in making this crown as no pins are really needed, though when desired a plate tooth can be used by soldering pins to tube and insuring position, but in this way investing would be necessary and would take more time. In baking the first time, the crown must be heated very carefully or the veneer will jump off. This is the only objection, for there being no pins there is no danger of checking.

This crown is easily and quickly made, and has the advantage of a perfect fit, and in the front of the mouth gives excellent service. When there is liability to root trouble the use of a hollow post is recommended, which should extend to cutting surface, with opening sealed with gutta-percha or something easily removed. If the crown breaks or splits from the post, this is easily and quickly repaired without interfering with the foundation. If the whole crown, including post, becomes loose, it is then treated as an ordinary pin crown and cemented in position.

In the use of bicuspids or molars I would suggest amalgam attachment, which is made by burning gold to the surface of attachment. The gold is that which is used for china decorating purposes, and is applied with small brushes, and then allowed to dry, presenting a brown appearance, then the tooth or whatever is coated is gradually heated till it shows a dull gold surface. To make an attachment cover the surface with very thin amalgam and burnish till the two metals unite, and then press the crown or filling into position and draw excess of mercury by wafering, using this method for porcelain fillings is sometimes very advantageous, making a non-leakable and almost indestructible piece of work. By cutting out the line of amalgam sufficiently to retain gold it will make a beautiful operation and overcome the objection to the possible dissolving of cement.

It is also by this means that a very fair imitation of gold filling is made on porcelain teeth, instead of the usual way of drilling a cavity with diamond points and filling with gold. That portion of tooth or block to be gilded is first touched with a corundum wheel, grinding off the glazed surface just the size required. The gold solution is thinned with spirits of turpentine and applied with a well-pointed pencil brush and allowed to harden, then heated till the metal shows itself. Too much heat will run the gold into small globules and render another coating necessary. After the gilding is made burnish the surface with an agate or steel point, which will make a bright and very good imitation filling, taking only a few minutes to accomplish, providing you have the necessary conveniences.

It is not particularly necessary to have a furnace for this work, as it only requires an ordinary blow-pipe heat. First protect the tooth from the flame by covering it with a little box or thimble made of plate platinum, leaving one end open to allow of seeing progress of work.

The "jacket crown is highly recommended as having many advantages. It has the combined qualities of many of our most reliable crowns, and yet retains its distinctiveness and originality. It can be used on any tooth in the mouth without destroying the pulp, allowing easy access and easily repaired if broken. It gives the strength and qualities of a gold cap without its conspicuousness. I have used it very successfully on split roots or where a canal has been reamed too large, and became useless for retention of pin crown; also where unnatural spaces exist between front teeth. It is equally serviceable where the tooth crown has been cut or brcken off flush with gum, by sinking screw posts and building up with amalgam the same as for the tube crowns, already described, excepting that the amalgam is built to point of post, making a sort of "dummy" tooth over which this crown is cemented. This has wonderful strength and natural appearance.

This crown is made by fitting a platinum band (gage

No. 30) to the root or prepared tooth, the same as with gold cap work, excepting that the joint must be overlapping instead of butting edges. The lingual and labial outlines of the adjacent teeth are marked on the tube as a guide to grind those portions away to gain shape instead of cutting with scissors. The lingual side is shaped with wheel on lathe, and a piece of the same gage platinum soldered to fit that portion by very small amount of pure gold. After trimming and fitting to root, the labial surface is ground thin enough to burnish and fit over the tooth, after which a thin veneer is fitted and held in position by the porcelain paste, carefully dried and baked the same as other crowns already mentioned. The crown is now fitted to the root and requirements noted, such as proper size, shape and thickness, and just where body is wanted. If the surface of veneer requires grinding it should be done at this stage, so that it will be glossed again by the last heat, which should be strong and of uniform degree. After final baking the platinum portion is polished and the crown is ready for adjusting, using thin cement and very gentle tapping with a pine stick. The crown should fit easy as there is danger of breaking the thin porcelain on the sides of the crown, or even checking the veneer itself.

The joints are lapped and made as close as possible, so that great and frequent heating will not entirely destroy the solder; excess of solder will flow over the surface of the platinum, causing porosity and destroy the porcelain adhesion, which may not be noticed at the time of operation, but will be more forcibly noted later on. The lingual surface is ground thin to give shape, so that there may be two flat surfaces to hold solder. When finished it gives the proper tooth contour.—*Items of Interest.*

